# *Helicobacter pylori* and Serum Magnesium Level in Hemodialysis Patients

**DOI:** 10.5812/jjm.11095

**Published:** 2014-02-01

**Authors:** Hamid Nasri

**Affiliations:** 1 Department of Nephrology, Division of Nephropathology, Isfahan University of Medical Sciences, Isfahan, IR Iran

**Keywords:** *Helicobacter pylori*, Magnesium, Hemodialysis, Chronic Renal Failure

Dear editor,

To understand, the aggravating factors of *Helicobacter pylori* in chronic renal failures, especially patients under treatment of dialysis, we conducted a research on 44 hemodialysis patients. At this study, we observed a significant positive correlation of anti-Helicobacter antibody with serum magnesium. The results of this investigation suggested the association of serum magnesium with the *H. pylori *infection (1). For a better investigation, we conducted another investigation on 94 type 2 diabetic patients who had a mean creatinine clearance of 62 ± 23 mL/min. In the second study, there was no significant correlation between serum anti-*H. pylori* specific antibody titer and serum magnesium level (2). It is obvious that magnesium ion acquisition is essential for *H. pylori* (1). We speculated that, the elevated serum magnesium level in hemodialysis patients and its higher concentration in the gastric mucosa may improve the gastric colonization of *H. pylori* in hemodialysis patients, but not in patients with various stages of kidney insufficiency who were not undergone dialysis yet (1, 2). To the best of our knowledge, these are the sole studies on the association of serum magnesium level and *H. pylori* in renal disease patients. Gastric complications are common in patients who underwent hemodialysis (3), and *H. pylori* infection is thought to have an important role in gastrointestinal disease in these patients (4-9), on the other hand magnesium is mainly excreted by kidney and magnesium metabolism is perturbed in patients with chronic kidney disease (10-14). Thus more studies are needed to prove the association of serum magnesium level and *H. pylori *infection in hemodialysis and finding the clinical relevance of our results. 
